# Correlation of age-of-onset of Atopic Dermatitis with *Filaggrin* loss-of-function variant status

**DOI:** 10.1038/s41598-020-59627-7

**Published:** 2020-02-17

**Authors:** S. P. Smieszek, S. Welsh, C. Xiao, J. Wang, C. Polymeropoulos, G. Birznieks, M. H. Polymeropoulos

**Affiliations:** Vanda Pharmaceuticals Inc., Washington, DC USA

**Keywords:** Disease genetics, Skin diseases, Genetics research, Risk factors

## Abstract

The genetic background of Atopic Dermatitis (AD) with chronic pruritus is complex. *Filaggrin* (*FLG*) is an essential gene in the epidermal barrier formation s. Loss-of-function (LOF) variants in *FLG* associated with skin barrier dysfunction constitute the most well-known genetic risk factor for AD. In this study, we focused on the frequency and effect of *FLG* loss-of-function variants in association with self-reported age-of-onset of AD. The dataset consisted of 386 whole-genome sequencing (WGS) samples. We observe a significant association between *FLG* LOF status and age-of-onset, with earlier age of onset of AD observed in the *FLG* LOF carrier group (p-value 0.0003, Wilcoxon two-sample test). We first tested this on the two most prevalent *FLG* variants. Interestingly, the effect is even stronger when considering all detected *FLG* LOF variants. Having two or more *FLG* LOF variants associates with the onset of AD at 2 years of age. In this study, we have shown enrichment of rare variants in the EDC region in cases compared with controls. Age-of-onset analysis shows not only the effect of the *FLG* and likely EDC variants in terms of the heightened risk of AD, but foremost enables to predict early-onset, lending further credence to the penetrance and causative effect of the identified variants. Understanding the genetic background and risk of early-onset is suggestive of skin barrier dysfunction etiology of AD with chronic pruritus

## Introduction

Atopic dermatitis (AD) is a chronic, inflammatory skin disease with an estimated prevalence of 7.3% in the US^1^. The genetic background of chronic pruritus in AD is complex. The heritability of AD is estimated to be around 75%^2^. Interestingly, when one or both parents have AD the risk of a child developing AD is higher than the risk of developing other atopic conditions, such as asthma and allergic rhinitis^3^. This suggests that there are genetic factors specific to AD beyond those for general atopy^4^.

One of the main functions of the skin is to act as a barrier between the individual and the environment, preventing water loss and at the same time preventing pathogen and allergen entry^5^. Skin barrier dysfunction is a key clinical feature of AD, as this facilitates penetration of allergens, immunological dysfunction, and consequently an increased risk of developing eczema^5,6^. The skin barrier dysfunction has been associated with the etiology of the itch-scratch cycle. Genes encoding skin barrier proteins have been shown to play a role in the heritability of AD^7,8^. *FLG* is the most studied gene in AD. Loss of function (LOF) variants resulting in aberrant *FLG* production, constitute the best-known AD gene-association and have been shown to predispose individuals to AD^5,8^. *FLG* initially synthesizes profilaggrin, which is then transformed to *FLG* monomers which interact with intermediate filaments in the stratum corneum (SC), causing such to aggregate into dense parallel arrays of macrofilaments. This promotes cellular compaction and keratin crosslinking in the SC, which forms a highly insoluble matrix that acts as a protective barrier^5,9^.

The disrupted skin barrier of individuals harboring *FLG* LOF variants is characterized by dry and fissured skin. LOF variants in *FLG* are associated with lower levels of natural moisturizing factors in AD^10^. This facilitates penetration of allergens, immunological dysfunction, and consequently an increased risk of developing eczema^5,6^. Two prevalent *FLG* LOF variants, p.R501* and p.S761fs were identified as causes of Ichthyosis vulgaris, (dry, thick, scaly skin), a common feature of moderate to severe AD^11,12^. The allelic frequency of these variants in European cohort with eczema were estimated to be 2.8% and 6.6% for p.R501* and p.S761fs respectively, with a combined frequency of 9.3%^13,14^. They demonstrate the strongest association with AD, at 18% and 48% for moderate and severe disease, respectively^15^. However, both the p.R501* and p.S761fs variants, as well as the other two prevalent variants in Europeans, p.S324* and p.R2447*, are highly uncommon in Asian populations^16–18^. Furthermore, most of the identified *FLG* null variants display a higher prevalence in individuals of Caucasian ancestry as compared to the African Americans (27.5% vs. 5.8%)^19^.

The genetic architecture of AD is likely a combination of common variants, but also rare LOF variants within the *FLG* and pathways involved^12^. Several rare *FLG* LOF variants have been identified across different populations. For example, two rare LOF variants not found in European groups were identified in Asian AD populations, p.S2554* and p.S1107fs (prevalence 4.2% and 1.4%, respectively)^16^. Two studies in Singaporean Chinese AD populations identified a total of 14 additional *FLG* LOF variants^20,21^.

Additional genes involved in skin barrier function are thought to have a potential role in AD. The epidermal differentiation complex (EDC) encodes proteins critical to the proper development of keratinocytes and normal formation of the skin barrier^22^. The proteins in the EDC come from three gene families with closely related functions: the cornified envelop precursor family, and the S100 protein family and the S100 fused type proteins (SFTP)^22,23^. *FLG*, which is located on chromosome 1q21, is a member of the SFTP family of the EDC^5^. Dysregulation of other EDC genes has also been implicated in AD^24,25^. Several profiling studies of the transcriptome have shown significant downregulation of EDC genes, such as *ivolucrin, loricrin* and *late cornified envelop 2B*, in AD^24–26^. For example, the deletion of the EDC member gene *Small Proline-Rich Protein 3* was shown to be associated with AD^27^. Another large-scale genome-wide association study of AD patients also identified multiple risk loci in genes involved in epidermal proliferation and differentiation, in addition to a strong signal at the *FLG* locus^28^.

Our work aimed to investigate the frequency and effect of rare *FLG* and EDC LOF variants on the age of onset, on the severity of the phenotype itself and on clinically relevant measures in AD. We also evaluated other potential risk loci within the entire EDC in patients with AD, participants of a clinical study, VP-VLY-686-2102 (also referred to as AD1 throughout this paper). Study AD1 was a randomized, double-blind, placebo-controlled, multicenter study of 168 patients (placebo or treatment) with chronic pruritus associated with AD. The genetic results from this study were then replicated in a subset of a second ongoing clinical study, EPIONE (also referred to as AD2 throughout this paper). Study AD2 was also a randomized, double-blind, placebo-controlled, ongoing multicenter study of patients with chronic pruritus associated with AD. We evaluate the effect of these variants on the age-of-onset of AD to further understand the penetrance and the consequences of these variants in the phenotype context.

## Results

### Incidence of FLG variants in AD patients

**Figure 1:**
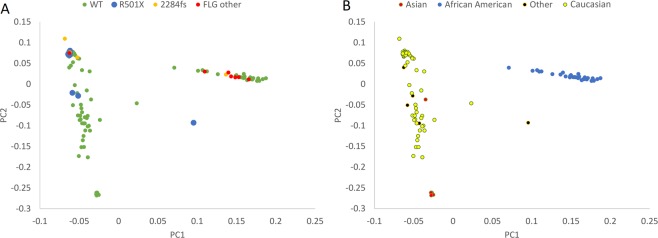
PCA plot displaying the population structure of AD1 cohort. (**A**) PCA plot colored by variant status. (**B**) PCA plot (WGS data) colored by ethnicity.

**Figure 2:**
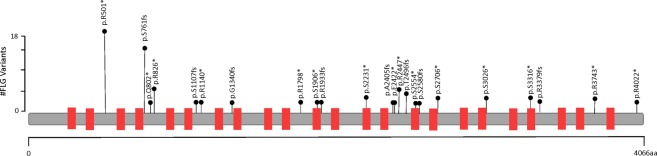
Lollipop plot displays the location and frequency of the identified variants in *FLG*. We observe an enrichment of rare LOF variants in *FLG* detected in AD patients, Red rectangles are *FLG* repeats.

**Figure 3:**
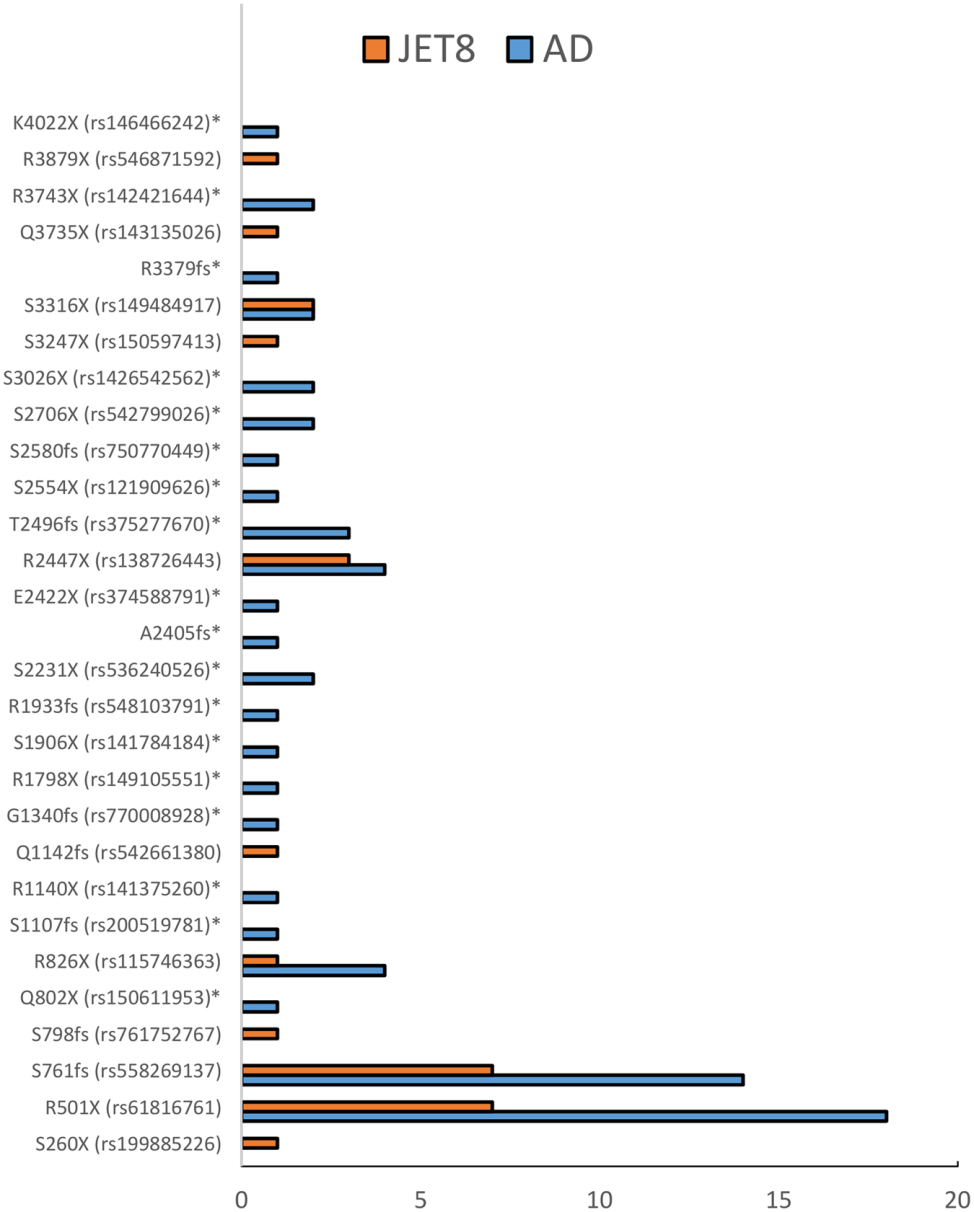
Comparison of variants detected in cases (AD – blue) and controls (JET8 - orange). We observe higher frequency of *FLG* variants in AD. The variants identified in AD cases not detected in controls are marked with a*.

**Figure 4:**
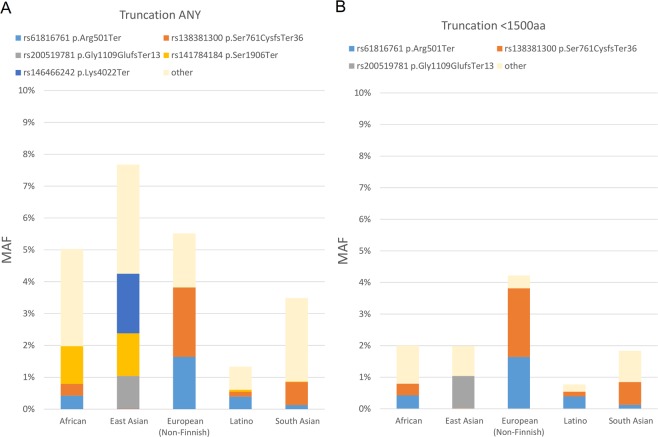
Prevalent *FLG* variants across ethnicities. MAF is displayed on the y-axis whereas the ethnicities are displayed on the x-axis. Panel A displays any truncation whereas Panel B displays truncation <1500 aa.

In clinical studyAD1, whole genome sequencing data was obtained from 116 subjects. In an ongoing study AD2 a subset of 270 patient samples were collected and whole genome sequenced. Figure 1 shows a PCA plot of the first study cohort 116/168, together with demographics and clinical characteristics of these patients provided in Supplementary Table 1. PCA was generated using WGS data, in order to examine population substructure of our studied cohort. Looking at panel B, we see enrichment of the two known variants in Caucasians and an interesting enrichment of other LOF *FLG* variants in the African American background. We investigated the incidence of all *FLG* LOF variants in the genomes of the clinical study patients, and compared them with the whole genome sequences of a control population of 316 healthy volunteers from clinical study 3107 (also referred to as JET8 throughout this paper). In AD1 study, 26 patients of the 116 (OR = 4.05, CI = 2.17–7.55, p < 0.0001) samples carried *FLG* LOF variant. In study AD2, 41 of the 270 sampled patients carried a *FLG* LOF variant (OR = 1.99, CI = 1.18–3.36, p = 0.0093). In the control population from the JET8 study, 21 of the 316 sampled patients harbored *FLG* LOF variants. The presence of *FLG* LOF variants in the combined population of AD1 and AD2 patients was significantly greater than the presence of these variants in the JET8 control population (OR = 2.95, CI = 1.76–4.93, p < 0.0001). The incidence of p.R501* variant alone was examined in the AD1 cohort, and was found in 10 of the 116 patients (MAF = 0.043). GnomAD’s allelic frequency total is 0.009 with the highest allelic frequency noted for European (Non-Finnish) population. The list of all the identified *FLG* LOF is present in Table 1 and displayed on Figs. 2 and 3. Figure 2 displays the location of individual LOF variant in both AD sets. We do observe a higher frequency of *FLG* LOF variants with many being rare singleton variants when compared to controls as displayed on Fig. 3. In addition, we have evaluated the presence of *FLG* population specific variants in gnomAD across ethnicities as shown on Fig. 4. Figure 4 shows the major frequency differences among populations especially when focusing the most actionable variants. Panel 4a. displays the frequent variants in the entire region whereas Panel B. displays truncating variants in the region <1500 aa. The motivation beyond examining truncation in the context of *FLG* LOFs was to explore physical truncation of the protein that would otherwise provide monomers as well as exploring the incidence of such.

**Table 1:** All LOF in *FLG* identified in the AD set.

Patient ID	Ethnicity	Gene	Transcript ID	NT change	AA change	RSziD	Chromosome	Start_Position	End_Position
1011019	Asian	*FLG*	NM_002016	c.C8117G	p.S2706*	rs542799026	1	152279245	152279245
1081007	Caucasian	*FLG*	NM_002016	c.2282_2285del	p.S761fs	rs558269137	1	152285077	152285077
1091012	African American	*FLG*	NM_002016	c.5799delG	p.R1933fs	rs548103791	1	152281563	152281563
1241019	Asian	*FLG*	NM_002016	c.C8117G	p.S2706*	rs542799026	1	152279245	152279245
1451005	Asian	*FLG*	NM_002016	c.7487delC	p.T2496fs	rs375277670	1	152279875	152279875
1471013	Caucasian	*FLG*	NM_002016	c.C1501T	p.R501*	rs61816761	1	152285861	152285861
1531004	Caucasian	*FLG*	NM_002016	c.10137delG	p.R3379fs	.	1	152277225	152277225
1601018	Caucasian	*FLG*	NM_002016	c.C1501T	p.R501*	rs61816761	1	152285861	152285861
1601033	Caucasian	*FLG*	NM_002016	c.2282_2285del	p.S761fs	rs558269137	1	152285077	152285077
1601033	Caucasian	*FLG*	NM_002016	c.C1501T	p.R501*	rs61816761	1	152285861	152285861
1601038	African American	*FLG*	NM_002016	c.7740_7741del	p.S2580fs	rs750770449	1	152279621	152279621
1621005	African American	*FLG*	NM_002016	c.C2476T	p.R826*	rs115746363	1	152284886	152284886
1631028	Caucasian	*FLG*	NM_002016	c.C9077G	p.S3026*	rs1426542562	1	152278285	152278285
1631028	Caucasian	*FLG*	NM_002016	c.C1501T	p.R501*	rs61816761	1	152285861	152285861
1711007	African American	*FLG*	NM_002016	c.C2404T	p.Q802*	rs150611953	1	152284958	152284958
1711027	Caucasian	*FLG*	NM_002016	c.C1501T	p.R501*	rs61816761	1	152285861	152285861
1721001	Caucasian	*FLG*	NM_002016	c.2282_2285del	p.S761fs	rs558269137	1	152285077	152285077
1721003	Caucasian	*FLG*	NM_002016	c.2282_2285del	p.S761fs	rs558269137	1	152285077	152285077
1721003	Caucasian	*FLG*	NM_002016	c.C1501T	p.R501*	rs61816761	1	152285861	152285861
42973718	Caucasian	*FLG*	NM_002016	c.2282_2285del	p.S761fs	rs558269137	1	152285077	152285077
42978887	Asian	*FLG*	NM_002016	c.7487delC	p.T2496fs	rs375277670	1	152279875	152279875
42989053	African American	*FLG*	NM_002016	c.C2476T	p.R826*	rs115746363	1	152284886	152284886
42989254	Caucasian	*FLG*	NM_002016	c.C1501T	p.R501*	rs61816761	1	152285861	152285861
43022549	African American	*FLG*	NM_002016	c.C7339T	p.R2447*	rs138726443	1	152280023	152280023
43022550	Caucasian	*FLG*	NM_002016	c.C1501T	p.R501*	rs61816761	1	152285861	152285861
43050811	African American	*FLG*	NM_002016	c.C5717A	p.S1906*	rs141784184	1	152281645	152281645
43055206	Caucasian	*FLG*	NM_002016	c.2282_2285del	p.S761fs	rs558269137	1	152285077	152285077
43062910	Caucasian	*FLG*	NM_002016	c.C1501T	p.R501*	rs61816761	1	152285861	152285861
43079509	African American	*FLG*	NM_002016	c.C6692A	p.S2231*	rs536240526	1	152280670	152280670
43099438	Caucasian	*FLG*	NM_002016	c.C7339T	p.R2447*	rs138726443	1	152280023	152280023
43099528	African American	*FLG*	NM_002016	c.C6692A	p.S2231*	rs536240526	1	152280670	152280670
43109908	Caucasian	*FLG*	NM_002016	c.C1501T	p.R501*	rs61816761	1	152285861	152285861
43116759	Caucasian	*FLG*	NM_002016	CNV_DELETION	CNV_DELETION	CNV_DELETION	1	152274652	152297680
43132454	Caucasian	*FLG*	NM_002016	c.C1501T	p.R501*	rs61816761	1	152285861	152285861
43134422	Caucasian	*FLG*	NM_002016	c.C1501T	p.R501*	rs61816761	1	152285861	152285861
43135174	Caucasian	*FLG*	NM_002016	c.C1501T	p.R501*	rs61816761	1	152285861	152285861
43148497	Caucasian	*FLG*	NM_002016	c.2282_2285del	p.S761fs	rs558269137	1	152285077	152285077
43179517	Caucasian	*FLG*	NM_002016	c.2282_2285del	p.S761fs	rs558269137	1	152285077	152285077
43198393	Caucasian	*FLG*	NM_002016	c.4020delA	p.G1340fs	rs770008928	1	152283341	152283342
43203074	African American	*FLG*	NM_002016	c.2282_2285del	p.S761fs	rs558269137	1	152285077	152285077
43221146	Caucasian	*FLG*	NM_002016	c.C1501T	p.R501*	rs61816761	1	152285861	152285861
43221147	Caucasian	*FLG*	NM_002016	c.C1501T	p.R501*	rs61816761	1	152285861	152285861
43260548	Caucasian	*FLG*	NM_002016	c.2282_2285del	p.S761fs	rs558269137	1	152285077	152285077
43297049	Caucasian	*FLG*	NM_002016	c.C1501T	p.R501*	rs61816761	1	152285861	152285861
43306754	African American	*FLG*	NM_002016	c.C2476T	p.R826*	rs115746363	1	152284886	152284886
5500365697	Caucasian	*FLG*	NM_002016	c.2282_2285del	p.S761fs	rs558269137	1	152285077	152285077
5502137729	African American	*FLG*	NM_002016	c.C9077G	p.S3026*	rs1426542562	1	152278285	152278285
5502362305	African American	*FLG*	NM_002016	c.C9947G	p.S3316*	rs149484917	1	152277415	152277415
5502362305	African American	*FLG*	NM_002016	c.C2476T	p.R826*	rs115746363	1	152284886	152284886
5502432960	African American	*FLG*	NM_002016	c.C5392T	p.R1798*	rs149105551	1	152281970	152281970
5504556880	Caucasian	*FLG*	NM_002016	c.C7339T	p.R2447*	rs138726443	1	152280023	152280023
5504556880	Caucasian	*FLG*	NM_002016	c.C1501T	p.R501*	rs61816761	1	152285861	152285861
5505347456	Caucasian	*FLG*	NM_002016	c.C1501T	p.R501*	rs61816761	1	152285861	152285861
5505609600	Asian	*FLG*	NM_002016	c.C7661G	p.S2554*	rs121909626	1	152279701	152279701
5506294081	Caucasian	*FLG*	NM_002016	c.2282_2285del	p.S761fs	rs558269137	1	152285077	152285077
5507409104	African American	*FLG*	NM_002016	c.2282_2285del	p.S761fs	rs558269137	1	152285077	152285077
5508687105	African American	*FLG*	NM_002016	c.C9947G	p.S3316*	rs149484917	1	152277415	152277415
5508688512	Caucasian	*FLG*	NM_002016	c.7487delC	p.T2496fs	rs375277670	1	152279875	152279875
5512912848	Caucasian	*FLG*	NM_002016	c.C11227T	p.R3743*	rs142421644	1	152276135	152276135
5514977857	African American	*FLG*	NM_002016	c.2282_2285del	p.S761fs	rs558269137	1	152285077	152285077
5515794176	Caucasian	*FLG*	NM_002016	c.C7339T	p.R2447*	rs138726443	1	152280023	152280023
5515801488	Asian	*FLG*	NM_002016	c.G7264T	p.E2422*	rs374588791	1	152280098	152280098
5515801488	Asian	*FLG*	NM_002016	c.7214_7215del	p.A2405fs	.	1	152280147	152280147
5515862528	Asian	*FLG*	NM_002016	c.A12064T	p.K4022*	rs146466242	1	152275298	152275298
5515996305	Asian	*FLG*	NM_002016	c.C3418T	p.R1140*	rs141375260	1	152283944	152283944
5516026753	Asian	*FLG*	NM_002016	c.C11227T	p.R3743*	rs142421644	1	152276135	152276135
5516124672	Asian	*FLG*	NM_002016	c.3321delA	p.S1107fs	rs200519781	1	152284041	152284041

### Enrichment of rare LOF variants in the EDC in AD patients

**Figure 5:**
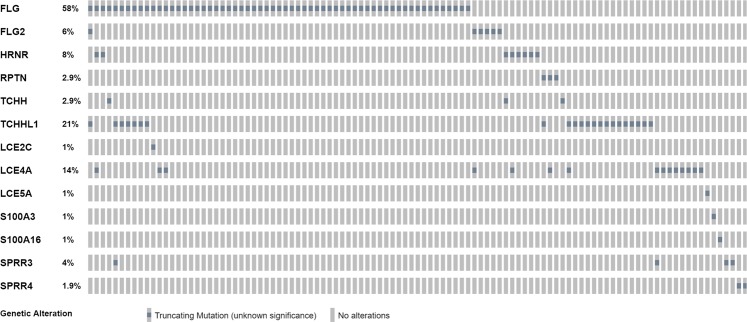
Percentage of LOF variants identified in both AD cohorts in the EDC (with high incidence of LOFs identified in the SFTP family). The empty gray boxes indicate no alteration whereas the filled gray boxes indicate alteration (LOF) present in a particular subject in that particular gene. The spatial distribution displays all the patients carrying at least a single variant in the queried genes.

The incidence of rare LOF variants in the EDC was investigated. Specifically, evolutionarily related members of the S100 fused type proteins (SFTP) family were evaluated, such as CRNN, *FLG2*, *HRNR* and *RPTN*, among other EDC genes all listed Table 2 and LOF in EDC displayed on Fig. 5. We investigated the frequency and effect of rare LOF (stopgain, frameshift, splicing) variants in the SFTP gene family in the AD1 population compared to the JET8 controls, as defined by a MAF < 5%. In the AD1 cohort, 45 of the 116, 38% AD patients carry a LOF variant (Table 2). In the AD2 cohort 75 of the 270, 27.7% AD patients carry a LOF variant. In the JET8 controls, 55 of the 316, 17% patients carried these LOF variants. Comparing these two results, the presence of these variants is significantly higher in the AD patients than the controls (OR = 2.18, CI = 1.51–3.13, p < 0.0001). Cumulative risks (OR and RR) were calculated for these rare variants as well, based on the presence of at least one deleterious allele. The relative risk (RR) for the rare SFTP LOF SNPs (n = 13) was found to be 2.27 (p = 0.0005). The OR was found to be 2.66 (p = 0.0007). In addition we computed the genetic risk score of these top variants shown on S Fig. 2. with a p-value of 0.00001. We were able to replicate the effect in the AD2 study cohort, OR 2.11, p-value = 0.022.

**Table 2:** ALL EDC LOFs identified in the AD set.

Patient ID	Ethnicity	*Gene*	Transcript ID	NT change	AA change	RSziD	Chromosome	Start_Position	End_Position
5500098961	Caucasian	*FLG2*	NM_001014342	c.5475_5478del	p.T1825fs	.	1	152324784	152324784
5508687105	African American	*FLG2*	NM_001014342	c.C5399G	p.S1800*	rs1349434249	1	152324863	152324863
1641003	African American	*FLG2*	NM_001014342	c.C5399G	p.S1800*	rs1349434249	1	152324863	152324863
1601037	African American	*FLG2*	NM_001014342	c.3940_3941insTA	p.T1314fs	rs567184084	1	152326321	152326321
1771002	Caucasian	*FLG2*	NM_001014342	c.C380A	p.S127*	rs373458772	1	152329882	152329882
43102554	African American	*FLG2*	NM_001014342	c.3940_3941insTA	p.T1314fs	rs567184084	1	152326321	152326321
1601035	African American	*HRNR*	NM_001009931	c.8467delA	p.S2823fs	rs1441063152	1	152185638	152185638
5508393600	Caucasian	*HRNR*	NM_001009931	c.C2299T	p.R767*	rs148459733	1	152191806	152191806
5505310928	Caucasian	*HRNR*	NM_001009931	c.C2299T	p.R767*	rs148459733	1	152191806	152191806
5500363216	Caucasian	*HRNR*	NM_001009931	c.C2299T	p.R767*	rs148459733	1	152191806	152191806
1711017	African American	*HRNR*	NM_001009931	c.C2299T	p.R767*	rs148459733	1	152191806	152191806
1061004	Caucasian	*HRNR*	NM_001009931	c.C2299T	p.R767*	rs148459733	1	152191806	152191806
42989053	African American	*HRNR*	NM_001009931	c.C2299T	p.R767*	rs148459733	1	152191806	152191806
43306754	African American	*HRNR*	NM_001009931	c.C2299T	p.R767*	rs148459733	1	152191806	152191806
1721003	Caucasian	*LCE2C*	NM_178429	c.C96A	p.C32*	rs4119577	1	152648587	152648587
5512683728	Caucasian	*LCE4A*	NM_178356	c.C9A	p.C3*	rs147765240	1	152681560	152681560
5509505040	African American	*LCE4A*	NM_178356	c.C9A	p.C3*	rs147765240	1	152681560	152681560
5506327056	African American	*LCE4A*	NM_178356	c.C9A	p.C3*	rs147765240	1	152681560	152681560
5502328576	African American	*LCE4A*	NM_178356	c.C9A	p.C3*	rs147765240	1	152681560	152681560
5502137729	African American	*LCE4A*	NM_178356	c.C9A	p.C3*	rs147765240	1	152681560	152681560
1681005	African American	*LCE4A*	NM_178356	c.C9A	p.C3*	rs147765240	1	152681560	152681560
1651002	African American	*LCE4A*	NM_178356	c.C9A	p.C3*	rs147765240	1	152681560	152681560
1641003	African American	*LCE4A*	NM_178356	c.C9A	p.C3*	rs147765240	1	152681560	152681560
1601035	African American	*LCE4A*	NM_178356	c.C9A	p.C3*	rs147765240	1	152681560	152681560
1511001	African American	*LCE4A*	NM_178356	c.C9A	p.C3*	rs147765240	1	152681560	152681560
1431001	African American	*LCE4A*	NM_178356	c.C9A	p.C3*	rs147765240	1	152681560	152681560
1371007	African American	*LCE4A*	NM_178356	c.C9A	p.C3*	rs147765240	1	152681560	152681560
5506294081	Caucasian	*LCE4A*	NM_178356	c.245_254del	p.G82fs	rs763134811	1	152681796	152681796
42989053	African American	*LCE4A*	NM_178356	c.C9A	p.C3*	rs147765240	1	152681560	152681560
43062909	African American	*LCE4A*	NM_178356	c.C9A	p.C3*	rs147765240	1	152681560	152681560
43181943	Caucasian	*LCE5A*	NM_178438	c.C235T	p.R79*	rs2282298	1	152484245	152484245
5502328576	African American	*RPTN*	NM_001122965	c.C2227T	p.R743*	rs184952075	1	152127348	152127348
1631025	African American	*RPTN*	NM_001122965	c.C2227T	p.R743*	rs184952075	1	152127348	152127348
5511537345	Caucasian	*RPTN*	NM_001122965	c.C1861T	p.Q621*	rs751330515	1	152127714	152127714
5515993537	Caucasian	*S100A16*	NM_001317007	c.C5G	p.S2*	rs137911276	1	153580623	153580623
43179516	Caucasian	*S100A3*	NM_002960	c.208delG	p.V70fs	rs576022937	1	153520255	153520255
1511001	African American	*SPRR3*	NM_001097589	c.189_205del	p.E63fs	rs746080074	1	152975685	152975685
1451006	African American	*SPRR3*	NM_001097589	c.189_205del	p.E63fs	rs746080074	1	152975685	152975685
1371005	African American	*SPRR3*	NM_001097589	c.189_205del	p.E63fs	rs746080074	1	152975685	152975685
43079509	African American	*SPRR3*	NM_001097589	c.189_205del	p.E63fs	rs746080074	1	152975685	152975685
43128537	African American	*SPRR4*	NM_173080	c.22_23insA	p.R8fs	rs201207143	1	152944388	152944388
43153173	African American	*SPRR4*	NM_173080	c.22_23insA	p.R8fs	rs201207143	1	152944388	152944388
5508393600	Caucasian	*TCHH*	NM_007113	c.C4309T	p.Q1437*	rs377677960	1	152081384	152081384
1611010	Caucasian	*TCHH*	NM_007113	c.C991T	p.Q331*	rs201930497	1	152084702	152084702
43050811	African American	*TCHH*	NM_007113	c.1delA	p.M1fs	rs748146582	1	152086555	152086555
5508457168	Caucasian	*TCHHL1*	NM_001008536	c.C2686T	p.Q896*	rs148113334	1	152057472	152057472
5516026753	Asian	*TCHHL1*	NM_001008536	c.C1966T	p.Q656*	rs150014958	1	152058192	152058192
5514715841	Caucasian	*TCHHL1*	NM_001008536	c.C1966T	p.Q656*	rs150014958	1	152058192	152058192
5511537345	Caucasian	*TCHHL1*	NM_001008536	c.C1966T	p.Q656*	rs150014958	1	152058192	152058192
5508719312	Caucasian	*TCHHL1*	NM_001008536	c.C1966T	p.Q656*	rs150014958	1	152058192	152058192
5508456912	Caucasian	*TCHHL1*	NM_001008536	c.C1966T	p.Q656*	rs150014958	1	152058192	152058192
5508456784	Caucasian	*TCHHL1*	NM_001008536	c.C1966T	p.Q656*	rs150014958	1	152058192	152058192
5507675024	Asian	*TCHHL1*	NM_001008536	c.C1966T	p.Q656*	rs150014958	1	152058192	152058192
5506589377	Caucasian	*TCHHL1*	NM_001008536	c.C1966T	p.Q656*	rs150014958	1	152058192	152058192
1711004	Caucasian	*TCHHL1*	NM_001008536	c.C1966T	p.Q656*	rs150014958	1	152058192	152058192
1601032	Caucasian	*TCHHL1*	NM_001008536	c.C1966T	p.Q656*	rs150014958	1	152058192	152058192
1481008	Caucasian	*TCHHL1*	NM_001008536	c.C1966T	p.Q656*	rs150014958	1	152058192	152058192
1301005	African American	*TCHHL1*	NM_001008536	c.C1966T	p.Q656*	rs150014958	1	152058192	152058192
1131007	Caucasian	*TCHHL1*	NM_001008536	c.C1966T	p.Q656*	rs150014958	1	152058192	152058192
1011018	Caucasian	*TCHHL1*	NM_001008536	c.C1966T	p.Q656*	rs150014958	1	152058192	152058192
5508687105	African American	*TCHHL1*	NM_001008536	c.C880T	p.Q294*	rs61749316	1	152059278	152059278
5502362305	African American	*TCHHL1*	NM_001008536	c.C880T	p.Q294*	rs61749316	1	152059278	152059278
1371007	African American	*TCHHL1*	NM_001008536	c.C880T	p.Q294*	rs61749316	1	152059278	152059278
43022549	African American	*TCHHL1*	NM_001008536	c.C1966T	p.Q656*	rs150014958	1	152058192	152058192
43099438	Caucasian	*TCHHL1*	NM_001008536	c.C1966T	p.Q656*	rs150014958	1	152058192	152058192
43079509	African American	*TCHHL1*	NM_001008536	c.C880T	p.Q294*	rs61749316	1	152059278	152059278
43099528	African American	*TCHHL1*	NM_001008536	c.C880T	p.Q294*	rs61749316	1	152059278	152059278

### Regional enrichment analysis reiterates the significance of the locus

In order to study the LOF variant set of the whole SFTP family of the EDC, an optimal unified sequence kernel association test (SKAT-O) was used. This test was applied to compare the WGS’s of AD1 cases versus healthy controls. Overall, a significant accumulation of rare variants in the EDC was observed in AD cases when compared to controls (p = 4.7E-20). This value is notably much lower than the association of *FLG* alone (p = 4.5E-6). Specifically, *en masse SKATO* analysis showed highly significant association with AD in *HRNR* looking beyond mere *FLG*, genes. Accumulation of rare LOF variants in the EDC yields a p-value of 4.7e-20, much lower than for *FLG* alone p-value of 4.5e-6 (LOF set comparing AD with controls) and reiterates the importance of looking for LOF variants extending beyond *FLG* itself.

### Association of variant status with age-of-onset of AD

We collected age of onset of AD information for the whole genome sequencing (WGS) samples also genotyped for 2 most prevalent *FLG* variants (p.R501* (rs61816761), p.S761fs (rs558269137)). The average age of onset in this cohort is 23.2 years of age (S Fig. 3. Histogram of age of onset). We observe a significant association between *FLG* LOF status and age-of-onset, with earlier age of onset of AD observed in the *FLG* LOF carrier group (p-value 0.0003, Wilcoxon two-sample test).The median age of onset for WT is 20 years of age, and the mean age of onset is 4.

**Figure 6:**
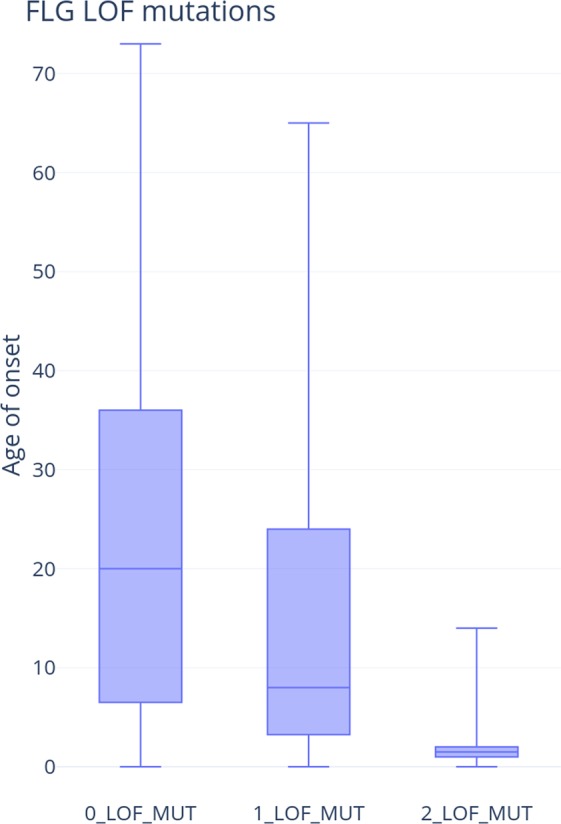
AD Age-of-onset box-plot of AD. We observe an earlier age-of-onset of AD in *FLG* LOF variant carriers. The effect is greater with higher frequency of FLG LOFs.

Interestingly, the effect is even stronger when considering all detected *FLG* LOF variants. Having two or more *FLG* LOF variants associates with onset of AD at 2 years of age (Wilcoxon Two-Sample Test). We observe a significant association between *FLG* LOF status and age-of-onset, with earlier age of onset of AD observed in the *FLG* LOF carrier group (*z*-score 3.95, *p-value* 0.00008) with the effect displayed on Fig. 6. The OR of having onset before 20 years of age in the AD population if the subject is a variant carrier is 8.9 (p-value 0.004). The OR of having onset before 5 years of age in the AD population if the subject is a variant carrier is 7.8 (p-value 0.0001). We have shown a significant enrichment of WGS for rare variants in the EDC region in cases compared with controls. Age-of-onset analysis shows not only the effect of the *FLG* and likely EDC variants in terms of heightened risk of AD, but foremost enables to predict early onset, lending further credence to the penetrance and causative effect of the identified variants.

## Discussion

Using screening methods that are dependent only on a subset of known *FLG* variants (p.R501*, p.S761fs etc.) we are missing out on ~30% of *FLG* LOF variation. Whole Genome Sequencing closes that gap of missing *FLG* impairment among AD patients. It is especially evident among non-European populations as shown in Figs. 1 and 4. With whole genome sequencing and focusing on all LOF variants we have shown that we can explain a higher proportion of patients as well as find other actionable variants (variants of well-established consequence on development and persistence of AD, that can guide diagnosis and likely therapeutic approaches) that are variable across populations. The same holds true when we expand to pathway based analyses and EDC complex, in example variants in *HRNR* and other SFTP family members.

It has been previously reported by Margolis *et al*., that subjects with an *FLG* variants were less likely (OR 0.54) to report as ‘symptom free’ in comparison with those without these variants. Interestingly, children in that cohort carrying the p.R501* variant (OR 0.44) were more likely to be non-responders to therapies^19^. Another interesting study reported by Koseki *et al*., investigating the effect of 8 LOF *FLG* variants, supports the notion that the effect of *FLG* LOFs variants is prominent during a very early stage of life^29^. Further evidence comes from another study focusing specifically on 2282del4 showing association with AD developed during infancy as reported by Rupnik *et al*.^30^. Furthermore, the authors showed an association with longer duration and more frequent hospitalization in this cohort of *FLG* variant carriers^30^. It is becoming more apparent that *FLG* and overall EDC, specifically SFTP variants predispose to risk of AD with effects differing across ages.

We used *FLG* and EDC status of variants and used that to correlate with age-of-onset and other clinical measures of AD and itch. The fact that age-of-onset is highly correlated with *FLG* status reflects upon the potential causes of the disorder itself, here likely skin barrier dysfunction as primary. The fact that frequency matters and amplifies the observed earlier onset lends credence to yet another hypothesis were the effect is intensified with number of causative variants.

There are several limitations of this work that may impact the conclusions as of course we extrapolate to EDC but there could be other ways of assigning pathway based categories that are involved in skin barrier function. Noteworthy is also the fact that we are not using controls evaluated for not having AD, so likely the magnitude of the effect would be even greater with such matched and screened for not having AD diagnosis set of controls. Nevertheless, it is likely that directionally our conclusions are valid and simply the magnitude may increase with differently matched controls. As we are accruing more subjects it will be very interesting to design a panel that would cover and work for different ethnicities as the majority of variants discussed in literature are applicable to Caucasian ancestry. It will also be relevant to quantify the effects of these variants on a translational basis looking at skin proteomics. In addition structural variants that are usually known to account for roughly 5% of the variation unexplained, should be evaluated, in this context to provide a more comprehensive outlook on the role of skin barrier genetics in AD.

## Conclusion

Understanding the genetic background and risk of early-onset is suggestive of skin barrier dysfunction etiology of the itch-scratch cycle present in early-onset AD. The pervasive effect of the variants would likely then manifest itself at an early age with AD phenotype of xerosis, and susceptibility to infection. Novel hence newly discovered LOF variants detected in this study expand the pool of risk loci. Whole-genome sequencing showed enrichment for rare variants in the EDC, specifically in the SFTP family, in AD patients compared to healthy controls. The strong association between these variants and AD suggests they may significantly affect a person’s risk of developing the disease. Further, these variants identified are often in genes that affect the skin barrier, lending evidence to the role of barrier dysfunction in the pathogenesis of AD. The novel LOF variants detected in this study help explain the missing heritability and etiology of AD and could serve as disease biomarkers, which could personalize treatment to improve disease outcomes. Adding the age component further reiterates the heritable component with an intensified effect and earlier onset observed at a young age and higher frequency of the LOF variants.

## Methods

### Clinical study design

The clinical studies VP-VLY-686-2102 and VP-VLY-686-3101 were multicenter, randomized, placebo-controlled, double-blind clinical studies in the United States. Participants provided written informed consent and were provided a copy of the signed consent form. All the studies were approved by the Advarra IRB located in Columbia, MD, USA and participants consented to participation in all the aspects of the studies. Methods were performed in accordance with the relevant guidelines and regulations, in adherence to the IRB approved protocol. Pharmacogenetic samples were taken at the screening visit for both studies. In EPIONE1, the self-reported age of onset was collected by the Medical History of Atopy questionnaire (developed by Vanda) at the first study visit. The study was divided into two phases: the screening phase and the evaluation phase (S Fig. 1).

Key study inclusion criteria included: chronic itch related to AD, defined as lasting 6 weeks or longer, that was refractory to previous treatment by patient history, average itch visual analog score (VAS) of greater than or equal to 70 mm out of 100 mm, and itch verbal response score (VRS) of greater than or equal to 3 on at least one of the past three days prior to randomization.

Study demographics were similar between treatment and placebo groups for sex, age, race and baseline itch and disease measures (S. Table 1).

### DNA quantification

Incoming nucleic acid samples are quantified using fluorescent-based assays (PicoGreen) to accurately determine whether sufficient material is available for library preparation and sequencing.

### DNA integrity

DNA sample size distributions are profiled by a Fragment Analyzer (Advanced Analytics) or BioAnalyzer (Agilent Technologies), to assess sample quality and integrity.

### Genotyping

At the NYGC, we run the HumanCoreExome 24v1.3 array for all human DNA samples that we sequence.

### WGS library preparation and sequencing, Truseq PCR-free (450 bp)

Whole genome sequencing (WGS) libraries were prepared using the Truseq DNA PCR-free Library Preparation Kit.

#### WGS Germline analysis part I

Whole Genome data were processed on NYGC automated pipeline. Paired-end 150 bp reads were aligned to the GRCh37 human reference (BWA-MEM v0.7.8) and processed with GATK best-practices workflow (GATK v3.4.0).

The mean coverage in the *FLG* region is 35.8, it reflects the samples average. The coverage for the *FLG* region tracks with the coverage for the whole genome. The coverage information per *FLG* region per sample is provided in the Supplementary File.

All high quality variants obtained from GATK were annotated for functional effects (intronic, intergenic, splicing, nonsynonymous, stopgain and frameshifts) based on RefSeq transcripts using Annovar (http://www.openbioinformatics.org/annovar/)^31^. Additionally, annovar was used to match general population frequencies from public databses (Exac, GnomAD, ESP6500, 1000 g) and was used to prioritize rare, loss-of-function variants.

#### Genomic data analysis part II

All variants in *FLG* and EDC were identified in whole genome sequencing data. The two most known genetic risk factors as defined by p.R501* (rs61816761), p.S761fs (rs558269137) were in addition verified with genotyping (S Fig. 4). All analyses were conducted in PLINK, MATLAB. In order to proceed with burden testing, we selected variants based on their quality, ancestry and pathogenicity, creating a subset of high-confidence variants. Variants that met these quality and pathogenicity filters were used for burden testing versus a. internal controls and b. Genome Aggregation Database (gnomAD).

## Supplementary information

Supplementary Material.

Supplementary Dataset 1.
