# Supplementary material for: Correlation of age-of-onset of Atopic Dermatitis with Filaggrin loss-of-function variant status
Source: Sci Rep. 2020 Feb 17;10:2721. doi: 10.1038/s41598-020-59627-7 (PMC7026049; doi:10.1038/s41598-020-59627-7)
Supplement: Supplementary file 1 — Supplementary Material. [file 41598_2020_59627_MOESM1_ESM.pdf]

Correlation of age-of-onset of Atopic Dermatitis with *Filaggrin* loss-of-function variant status

Smieszek SP[1], Welsh S[1], Xiao C[1], Wang J[1], Polymeropoulos C[1], Birznieks G[1], Polymeropoulos MH[1]

[1] Vanda Pharmaceuticals Inc., Washington, DC

Corresponding Author: Sandra.Smieszek@vandapharma.com

S. Table 1

Table 1. Study Demographics

All Randomized Subjects	Tradipitant (N=84)	Placebo (N=84)	Total (N=168)
Gender - n (%)			
Male	32 (38.1)	31 (36.9)	63 (37.5)
Female	52 (61.9)	53 (63.1)	105 (62.5)
Age (years)			
Mean (min,max)	41 (18,66)	39 (18,64)	40 (18,66)
Race - n (%)			
White	49 (58.3)	57 (67.9)	106 (63.1)
Black or African American	24 (28.6)	18 (21.4)	42 (25.0)
Asian	6 (7.1)	5 (6.0)	11 (6.5)
American Indian or Alaska Native	0	1 (1.2)	1 (0.6)
Native Hawaiian or Other Pacific Islander	2 (2.4)	0	2 (1.2)
Other	3 (3.6)	3 (3.6)	6 (3.6)
Itch VAS			
Average *Mean (SD)*	81.3 (10.8)	80.3 (8.7)	80.8 (9.8)
Worst *Mean (SD)*	85.1 (10.0)	82.7 (10.0)	83.9 (10.0)
SCORAD			
Mean (SD)	47.4 (13.0)	45.7 (13.7)	46.5 (13.3)

S. Figure 1

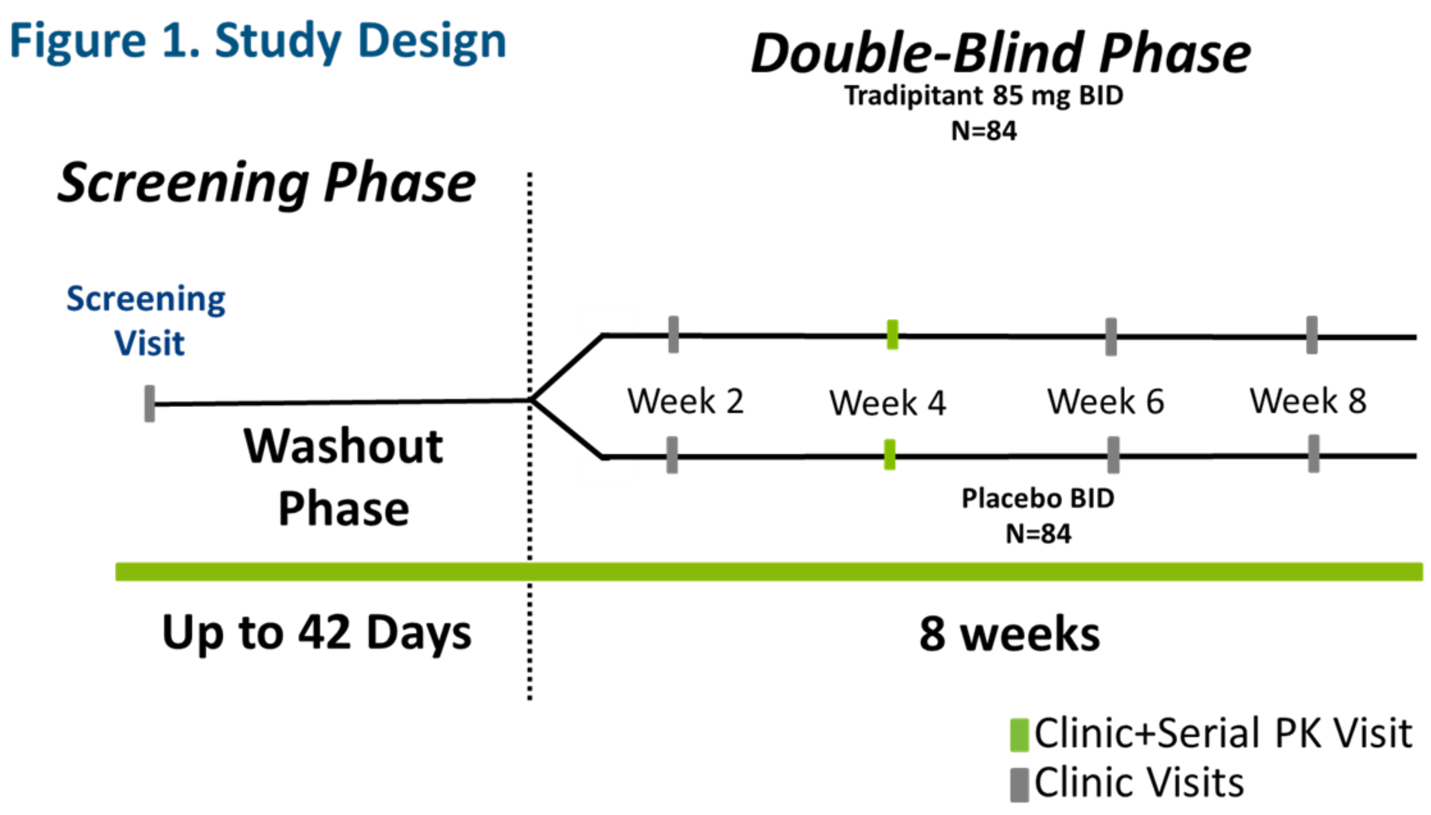
Figure 1. Study Design
Double-Blind Phase
Tradipitant 85 mg BID
N=84
Screening Phase
Screening
Visit
Week 2
Week 4
Week 6
Week 8
Washout
Phase
Placebo BID
N=84
Up to 42 Days
8 weeks
Clinic+Serial PK Visit
Clinic Visits

S. Figure 2

CHR	BP	VARID	GENE	VARTYPE	AA CHANGE
1	152059278	1:152059278[b37]A,G	TCHHL1	stopgain	NM_001008536:c.C880T:p.Q294X
1	152086555	1:152086555[b37]A,AT	TCHH	frameshift	NM_007113:c.1delA:p.M1fs
1	152191806	1:152191806[b37]A,G	HRNR	stopgain	NM_001009931:c.C2299T:p.R767X
1	152279874	1:152279874[b37]T,TG	FLG	frameshift	NM_002016:c.7487delC:p.T2496fs
1	152280023	1:152280023[b37]A,G	FLG	stopgain	NM_002016:c.C7339T:p.R2447X
1	152280670	1:152280670[b37]G,T	FLG	stopgain	NM_002016:c.C6692A:p.S2231X
1	152281645	1:152281645[b37]G,T	FLG	stopgain	NM_002016:c.C5717A:p.S1906X
1	152283341	1:152283341[b37]G,GT	FLG	frameshift	NM_002016:c.4020delA:p.G1340fs
1	152284886	1:152284886[b37]A,G	FLG	stopgain	NM_002016:c.C2476T:p.R826X
1	152285076	1:152285076[b37]C,CACTG	FLG	frameshift deletion	NM_002016:c.2282_2285del:p.S761fs
1	152285861	1:152285861[b37]A,G	FLG	stopgain	NM_002016:c.C1501T:p.R501X
1	152323658	1:152323658[b37]T,TGTGA	FLG2	frameshift	NM_001014342:c.6600_6603del:p.T2200fs
1	152326321	1:152326321[b37]G,GTA	FLG2	frameshift	NM_001014342:c.3940_3941insTA:p.T1314fs

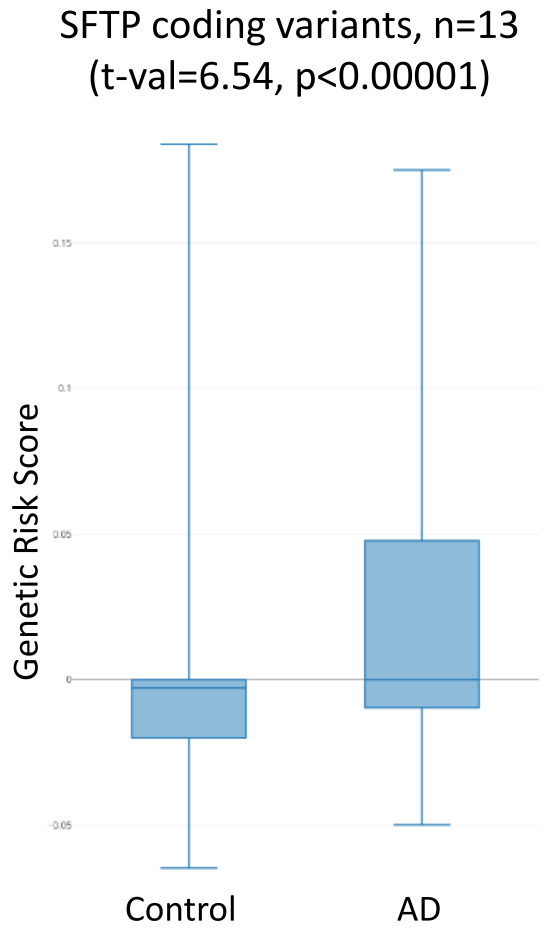

S.Figure 3

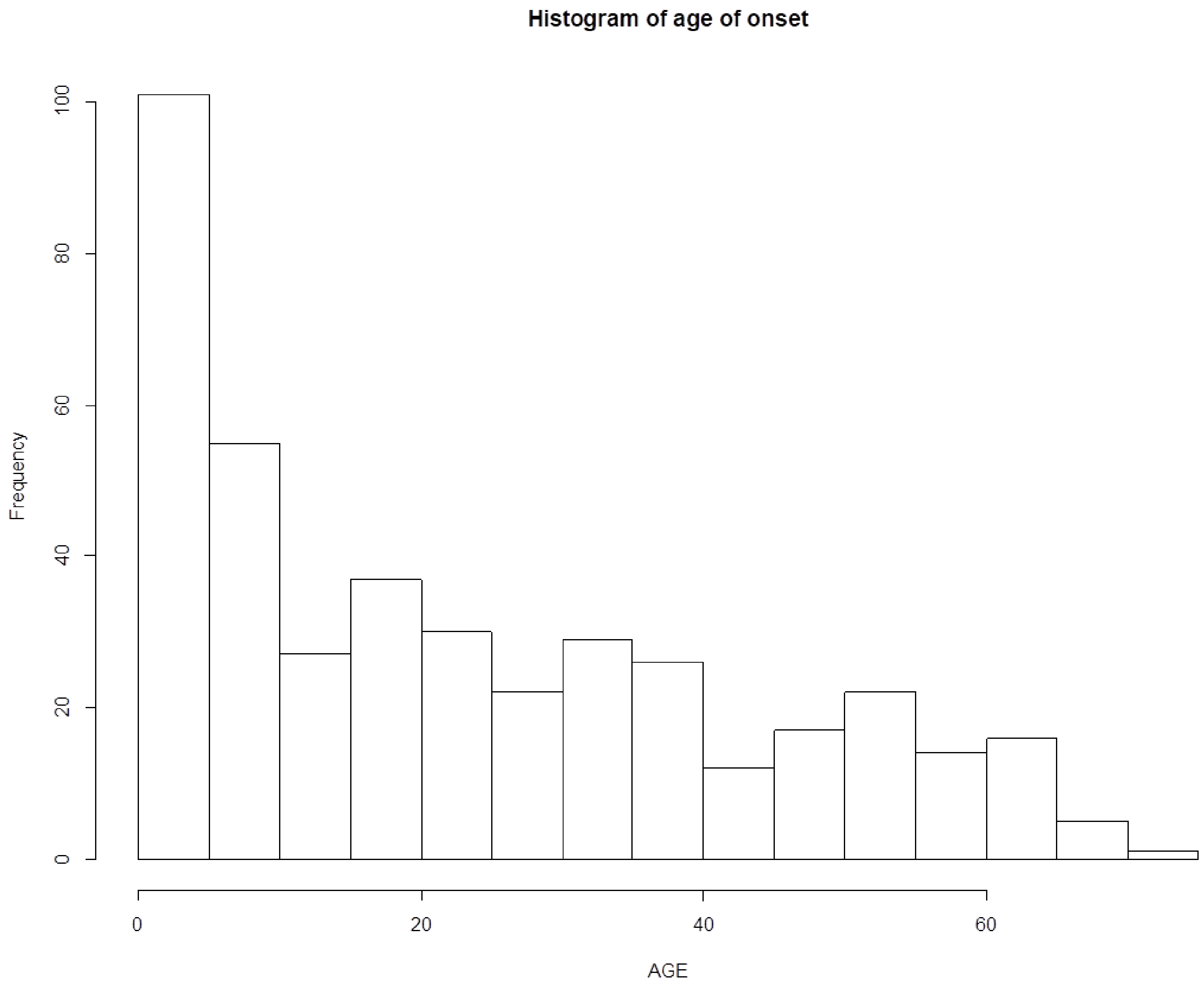

Histogram of age of onset
Frequency
0
20
40
60
80
100
0
20
40
60
AGE

S.Figure 4

For 501X R and F respectively x 2

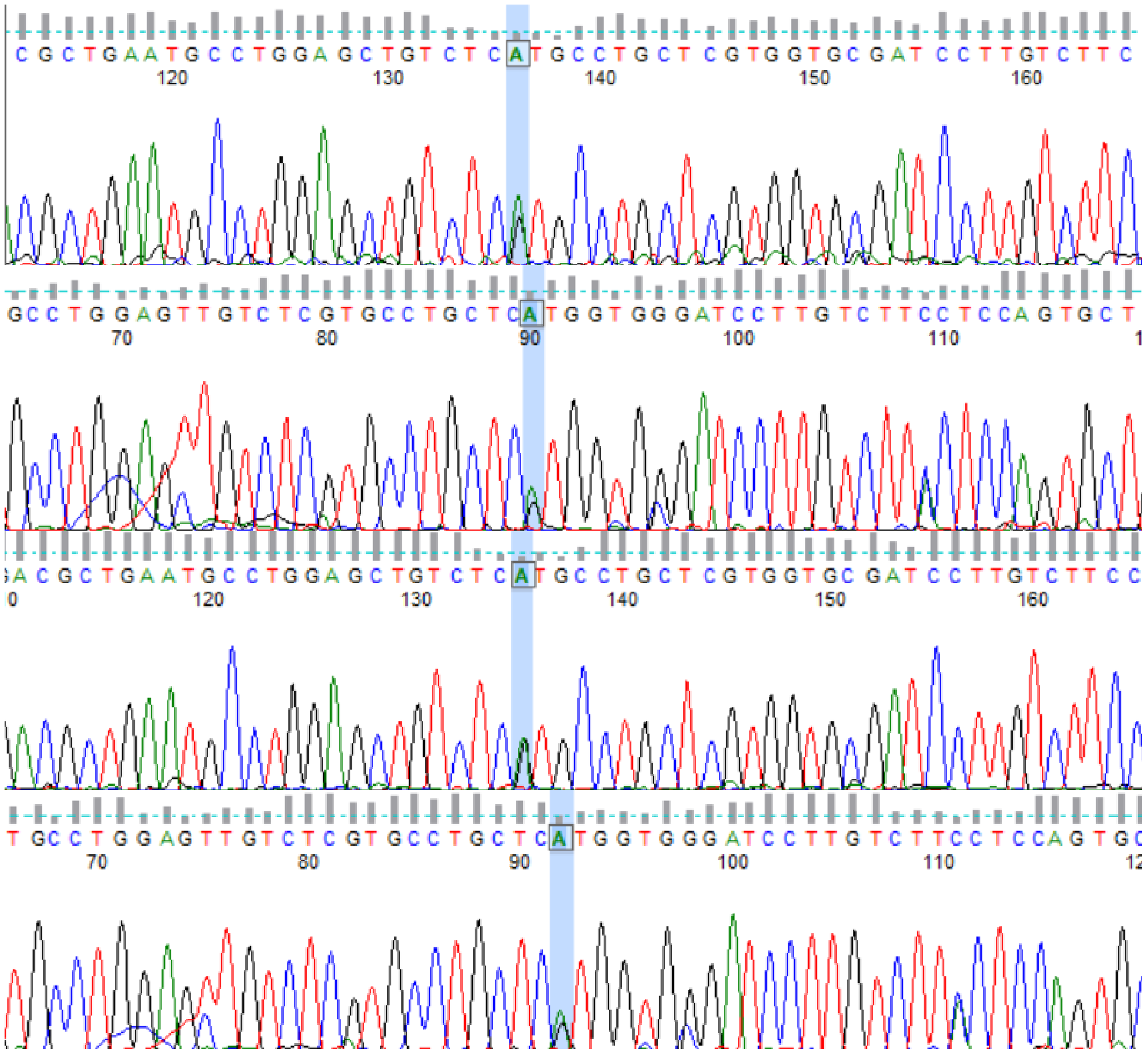

Former figure 2, now replaced with lollipop plot

Enrichment of rare FLG LOF variants in FLG in AD patients (2 studies) and control population

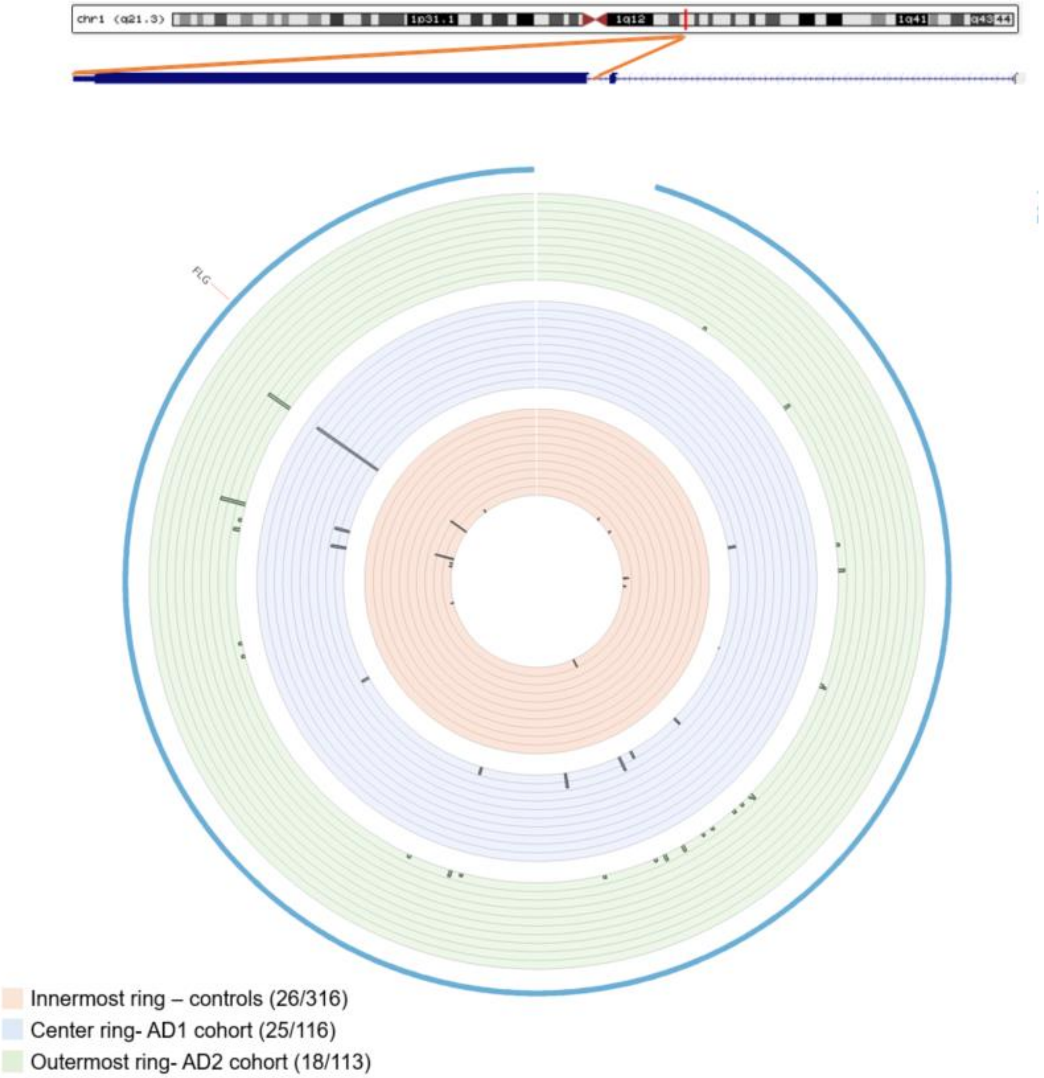

		OR	p-value
AD1	25/116	3.06	0.0002
AD2	18/113	2.11	0.022
